# Integration of 2D and 3D Imaging Descriptors with Large Language Models for Assessing Pediatric Foreign-Body Aspiration Risk

**DOI:** 10.3390/children13050684

**Published:** 2026-05-16

**Authors:** Dario Gregori, Cinzia Anna Maria Papappicco, Dario Vucinic, Chiara Giraudo, Azra Ibrisevic, Alen Harcinovic, Šekib Umihanić, Fuad Brkic, Solidea Baldas, Giulia Lorenzoni, Honoria Ocagli

**Affiliations:** 1 Unit of Biostatistics, Epidemiology and Public Health, Department of Cardiac, Thoracic, Vascular Sciences and Public Health, University of Padova, 35131 Padova, Italy; cinziaannamaria.papappicco@ubep.unipd.it (C.A.M.P.); dario.vucinic@ubep.unipd.it (D.V.); giulia.lorenzoni@unipd.it (G.L.); honoria.ocagli@unipd.it (H.O.); 2 BIOSTAT-X Biostatistics & AI for Biomedical Discovery, Pediatric Research Institute (IRP) “Città della Speranza”, 35127 Padova, Italy; 3 PhD Program in Translational Specialistic Medicine “G.B. Morgagni”, Curriculum “Nursing and Health Sciences”, University of Padova, 35131 Padova, Italy; 4 Unit of Advanced Clinical and Translational Imaging, Department of Cardiac, Thoracic, Vascular Sciences and Public Health, University of Padova, 35128 Paddova, Italy; chiara.giraudo@unipd.it; 5 Department of Otorhinolaryngology, University Clinical Center Tuzla, 75000 Tuzla, Bosnia and Herzegovina; azra.ibrisevic@ukctuzla.ba (A.I.); alen.harcinovic@ukctuzla.ba (A.H.); sekib.umihanic@ukctuzla.ba (Š.U.); fuad.brkic@ukctuzla.ba (F.B.); 6 Protecting Children Association (Prochild) Onlus, 34129 Trieste, Italy; solidebaldas@prochild.eu

**Keywords:** foreign-body aspiration, pediatric airway, 3D/2D imaging analysis, radiomic features, large language models, aspiration risk assessment, injury prevention, pediatric safety

## Abstract

**Highlights:**

**What are the main findings?**

- Quantitative 2D and 3D morphometric descriptors, combined with LLM-based interpretation, enable a structured and informed assessment of pediatric foreign-body aspiration risk.
- Object morphology, including shape, sharpness, and orientation, substantially influences both where an aspirated foreign body lodges in the pediatric airway and the severity of the resulting injury, beyond traditional size-based criteria.

**What are the implications of the main findings?**

- For practicing pediatricians, incorporating shape-related metrics into hazard assessment may complement existing size-based safety standards and improve the bedside identification of high-risk objects.
- The proposed framework offers a basis for developing interpretable tools to support prevention strategies, product design, caregiver education, and clinical risk assessment.

**Abstract:**

**Background/Objectives**: Foreign-body aspiration (FBA) is a common and largely preventable pediatric emergency, yet current safety standards and risk assessments rely predominantly on object size and on anecdotal descriptions and bronchoscopy findings. We propose a clinically oriented proof-of-concept workflow that combines high-resolution three-dimensional (3D) scanning and calibrated two-dimensional (2D) imaging of retrieved objects with radiomic shape descriptors and large language model (LLM) reasoning to support aspiration risk assessment and guide prevention. **Methods**: Objects were obtained from the Susy Safe registry and historical series from the University Clinical Centre Tuzla. Each object was digitized with 3D scanning and photographed with a ruler. Morphometric descriptors—including volume, surface area, sphericity, elongation, flatness, curvature and convexity—were computed from stereolithography (STL) meshes; silhouette area, perimeter and Feret diameters were extracted from 2D photographs. Normative airway dimensions from radiographic and computed tomography (CT) studies provided anatomical context. A sharp, irregular metallic object recovered from a child’s laryngo-tracheal tract served as an illustrative case. **Results**: The object’s major axis approximated the anteroposterior glottic diameter, suggesting potential traversal when longitudinally oriented, whereas its irregular shape increased the likelihood of mucosal laceration and lodging. LLM-based synthesis provided a structured narrative interpretation consistent with a high-risk profile and highlighted preventive implications. **Conclusions**: Combining 2D/3D morphometry with LLM reasoning provides objective assessment of FBA hazards and may support safer product design, injury-prevention policies, and caregiver education.

## 1. Introduction

Foreign-body aspiration is a leading cause of injury and death in children under three years of age and remains a major global public-health concern [1]. In the United States alone, choking and foreign-body aspiration (FBA) are responsible for more than 100 deaths each year among children under five years of age, underscoring the magnitude of this injury at the population level [2]. The consequences include preventable emergency events and hospital admissions, a non-negligible risk of severe or fatal outcomes, and a substantial impact on health systems, which has led to repeated targeting through international injury-prevention policies, product-safety regulations, and caregiver education campaigns [3,4].

Young children are particularly vulnerable because they explore their environment orally and have immature protective reflexes; even small objects such as peanuts, pins or toy fragments may enter the airway [5]. The clinical outcome depends on where the object lodges: slender, sharp objects tend to travel deeper into the bronchial tree and may injure the cricoid membrane [5], whereas larger or irregular objects may obstruct the glottis or esophagus [5]. In addition to mechanical features, the biological nature of the object plays an important role in the evolution of airway obstruction. Organic foreign bodies—such as peanuts, beans, seeds, or other vegetal materials—can absorb airway secretions and swell over time, so that an initially partial obstruction may progress to a complete one, with worsening hypoxia and inflammatory mucosal changes [6].

Understanding how an object’s morphology interacts with airway anatomy could improve risk prediction, given the largely preventable nature of the phenomenon, yet current guidelines classify hazards mainly by size—for instance, the U.S. Consumer Product Safety Commission defines a small part as any object fitting entirely into a test cylinder approximating the child’s throat [1]. However, this approach ignores relevant shape properties. The biomechanics of aspiration suggest that object morphology plays a pivotal role in determining airway behavior. Factors such as aspect ratio, surface roughness, curvature and center-of-mass distribution influence whether an object accelerates through the glottic aperture, rotates within the subglottic space or becomes lodged at bifurcation points of the bronchial tree. Sharp-edged objects exert focal pressure that predisposes to mucosal laceration, whereas smooth spherical items may travel deeper but with less injury [5]. These interactions are further governed by the unique dimensions and compliance properties of the pediatric airway, which differ substantially from adult anatomy [7,8]. Quantifying such geometric properties through radiomic descriptors provides a principled way to model these biomechanical relationships, potentially enabling a more nuanced and physiologically grounded classification of choking hazards [9,10]. Previous studies have associated object type and gross shape characteristics with different lodging sites and clinical presentations in pediatric FBA; however, these associations have been predominantly described using qualitative or categorical descriptors [5,11]. Advances in optical scanning and image processing allow precise three-dimensional (3D) and two-dimensional (2D) digitization of physical objects. Morphometric descriptors such as volume, surface area, sphericity and curvature—commonly used in radiomics—can quantify features relevant to aspiration risk [11]. Large language models (LLMs) offer a complementary capability: they can assimilate heterogeneous quantitative descriptors with medical literature to produce human-readable risk assessments.

From a clinical management perspective, the cornerstone of treatment for confirmed airway foreign bodies remains endoscopic extraction. Rigid bronchoscopy under general anesthesia has historically been the procedure of choice in pediatric airway foreign body removal, particularly in emergency settings and for large or impacted objects, while flexible bronchoscopy is increasingly used in selected cases and when the location or nature of the object favors a less invasive approach [12]. Reported success rates of bronchoscopic extraction in pediatric series typically exceed 95%, with no significant difference in overall efficacy between the two techniques, although rigid bronchoscopy shows a lower rate of respiratory complications and flexible bronchoscopy a lower risk of desaturation [12,13]. The most frequently reported complications include laryngeal edema, bronchospasm, transient desaturation, mucosal bleeding, and, less commonly, pneumothorax or subcutaneous emphysema, while mortality is consistently reported below 1% in contemporary high-volume centers [1,13]. Knowledge of the typical morphology and lodgment patterns of aspirated objects can therefore inform not only prevention but also procedural planning and the choice of the most appropriate extraction technique.

In this study, we present a complete workflow in which 3D scanning, 2D imaging and radiomic feature analysis of a recovered foreign body are coupled with LLM reasoning. Our goals are to demonstrate the feasibility of this approach, quantitatively compare object descriptors with normative pediatric airway dimensions, and discuss implications for prevention. We situate our analysis within the context of the Tuzla experience—an institutional series of 772 bronchoscopies performed between 1971 and 2013, with a mortality rate below 1% [1]—and the broader Susy Safe registry.

## 2. Materials and Methods

### 2.1. Data Sources

#### 2.1.1. Clinical Datasets

We drew on two complementary data sources. The Susy Safe registry, a multicenter database of foreign body injuries in children under 14 years, provided access to 500 physical objects, digitized via high-resolution 3D scanning [11]. The Tuzla series, an institutional dataset from the University Clinical Centre Tuzla in Bosnia and Herzegovina, includes 772 children who underwent rigid bronchoscopy for airway foreign bodies between 1971 and 2013. Overall mortality in this series was 0.785%, and no deaths occurred in the last 15 years of follow-up [1]. Though the full Tracheobronchial foreign bodies in children study is behind a paywall [14] the same dataset is described in an open-access mortality analysis [1]. Normative airway dimensions used for comparison in this study were extracted from radiographic and computed tomography (CT) studies, as summarized in Section 2.4.

#### 2.1.2. Case Study

**Figure 1:**
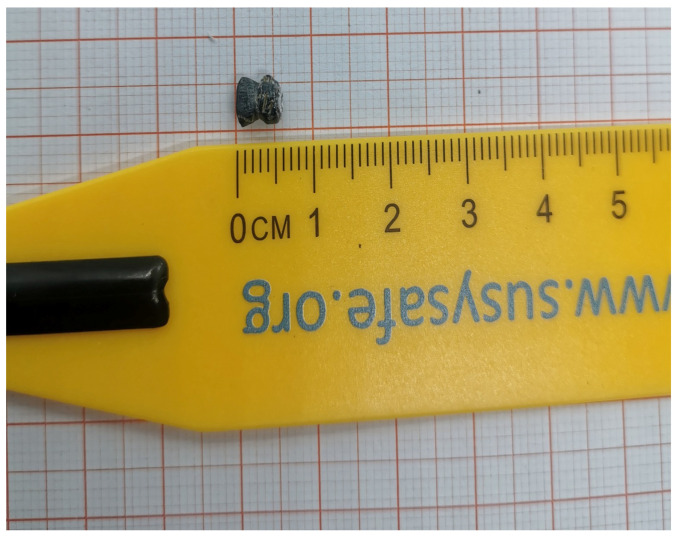
Photograph of the case object alongside a descriptor ruler used for spatial calibration of the 2D image analysis. The object is a small metallic fragment removed from a child’s airway. The scale bar indicates centimeters. The ruler provides the reference scale used to convert pixel measurements into physical dimensions during silhouette extraction and planar morphometric descriptor computation.

**Figure A1:**
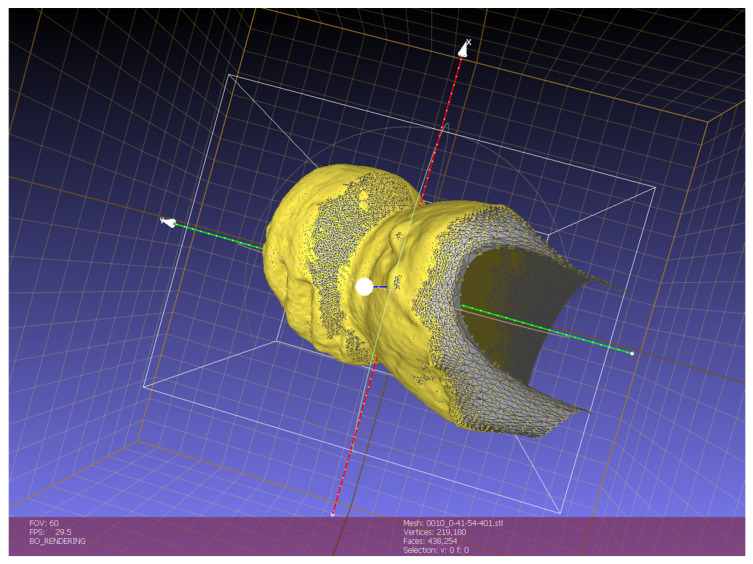
3D surface representation of the retrieved foreign body obtained through structured-light scanning and used for volumetric and shape analysis.

**Figure A2:**
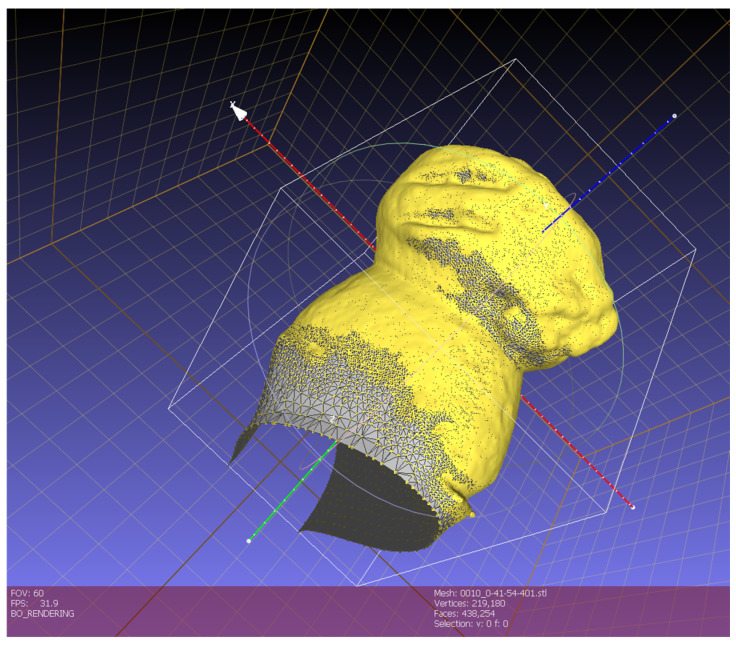
Calibrated 2D image and extracted silhouette used for planar morphometric analysis, including Feret diameters and shape descriptors.

A metallic foreign body retrieved from a child’s laryngo-tracheal tract served as the case object. It was scanned ex vivo using a structured-light 3D scanner with sub-millimetric nominal resolution that outputs a triangulated stereolithography (STL) mesh (Appendix A, Figure A1 and Figure A2). The scanning procedure followed the standard manufacturer protocol for small rigid objects, with the item placed on a neutral matte support to minimize reflections. The structured-light system was calibrated prior to acquisition according to the manufacturer’s standard procedure (calibration target supplied with the device); 3D geometric accuracy was further cross-checked at the analysis stage against independent volume and surface-area computations from the numpy-stl library. A photograph of the object placed beside a descriptor ruler was captured with a smartphone camera under standard indoor lighting, with the object resting on a flat surface and the camera approximately perpendicular to the support to minimize perspective distortion (Figure 1). Spatial calibration was performed in-image using the ruler tick marks, so that the metric accuracy of the 2D analysis does not depend on the specific camera model. Both files were anonymized prior to analysis and processed offline.

#### 2.1.3. 3D Morphometric Analysis

The STL file of the case object was parsed using a custom Python 3.11.x script. Each triangular face was read to extract vertex coordinates; duplicate vertices were collapsed. The object volume V was computed by summing the signed tetrahedral volumes formed between each triangular face and the origin; this method yields the exact volume for closed meshes. Surface area A was calculated as the sum of individual triangle areas. Principal component analysis (PCA) of the vertex coordinates provided the object’s principal axes. The square roots of the eigenvalues gave the major ($L_{1}$), intermediate ($L_{2}$) and minor ($L_{3}$) dimensions. PCA eigenvalues represent the variance of the point distribution along the principal axes and do not directly correspond to the physical dimensions of the object. In this framework, PCA is therefore used to determine the principal orientation of the reconstructed object, while the physical dimensions $L_{1}$, $L_{2}$, and $L_{3}$ are computed as the spatial extent of the point cloud projected along each principal axis (difference between the maximum and minimum coordinates along that axis). From these axes we derived dimensionless descriptors of shape, namely elongation and flatness, which summarize how rod-like and how flattened the object is. Elongation was defined as

(1) $$e=\sqrt{1-{{(L}_{3}/L_{1})}^{2}}$$

and flatness as

(2) $$f=\sqrt{1-{{(L}_{2}/L_{1})}^{2}}$$

both range from 0 for a perfectly spherical object to values closer to 1 for increasingly elongated or flattened shapes. In this context, the flatness descriptor is intended to capture the reduction in the intermediate dimension relative to the major axis within the overall object geometry. Although alternative definitions relate flatness to ratios involving the minor and intermediate axes, the adopted formulation emphasizes descriptor anisotropy and complements the elongation descriptor derived from the PCA-based principal axes. Sphericity ψ was calculated using the standard radiomics formula

(3) $$\psi ={\left(36{\pi V}^{2}\right)}^{1/3}/A$$

which equals 1 for a perfect sphere [9,10]. A convexity ratio was obtained as the ratio between the object volume and the volume of its convex hull. Surface roughness and sharpness were evaluated by computing dihedral angles between adjacent triangular faces; edges with dihedral angles below 90° were counted as sharp.

#### 2.1.4. 2D Image Analysis

The photograph of the object and ruler was converted to grayscale for 2D morphometric analysis. Because a single planar photograph captures the projection of the object onto the image plane, the resulting 2D descriptors reflect the silhouette of the object in the specific orientation in which it was placed for image acquisition rather than its full three-dimensional extent; this distinction is relevant for the interpretation of 2D versus 3D descriptors. The yellow ruler provided a calibration: tick marks separated by 5 cm were detected, yielding a scale of approximately 0.0469 mm per pixel. The object silhouette was then extracted by thresholding the dark region, applying morphological opening to remove noise and retaining the largest connected component as the object. Area and perimeter were measured in pixels and converted to mm^2^ and mm using the calibration factor. Feret diameters were computed using a rotating-calipers algorithm to find the maximum and minimum distances across the silhouette. Aspect ratio was defined as

(4) $$d_{min}/d_{max}$$

Circularity was calculated as

(5) $$4\pi A/P^{2},$$

where A is the silhouette area and P its perimeter [15]. Solidity was defined as the ratio between the silhouette area and the area of its convex hull, providing a measure of concavity [15]. Corner sharpness was estimated by fitting osculating circles along the contour; the smallest radius of curvature was recorded.

### 2.2. Normative Airway Dimensions

Several studies quantified pediatric airway dimensions. Plain radiographic measurements in children up to 4 years reported mean anteroposterior and transverse diameters of the glottis (9.8 mm × 3.4 mm), subglottis (8.5 mm × 5.6 mm) and cricoid (7.4 mm × 6.8 mm). Corresponding cross-sectional areas were 26.5, 38.1 and 40.5 mm^2^ [7]. CT and magnetic resonance (MR) imaging studies found similar anteroposterior diameters: 7–11 mm at the glottis and 8–9 mm in the subglottis/trachea for children under 2 years [8]. A clinical protocol from the University of Iowa notes that each 1 mm reduction in airway diameter (e.g., from 6 mm to 5 mm) halves the cross-sectional area and doubles resistance, highlighting the criticality of small differences [16]. Current paediatric endotracheal tube guidelines recommend internal diameters of 4.0–4.5 mm (outer diameters 5.3–6.0 mm) for 2-year-olds; ultrasonography studies report median proximal tracheal diameters of 7.3 mm in boys and 6.5 mm in girls and a mean cricoid diameter of 7.41 mm [17]. These normative values were used to contextualize the case object.

### 2.3. Validation of Calculations

To ensure reproducibility, the 3D descriptors were cross-checked using multiple approaches. Volume and surface area calculations were validated against numpy-stl outputs on a subset of objects from the Susy Safe database (differences < 0.1%). Principal axes were compared with inertia tensor computations, yielding differences in length estimates < 2%. For the 2D image, the calibration was tested by measuring known distances on the ruler (0–5 cm), which produced consistent scales across repeated segments. The segmented silhouette area was cross-validated by manual planimetry performed by two independent observers (disagreement < 5%). These procedures provide confidence that the reported descriptors are accurate. All computational analyses were implemented using custom scripts in R 4.3.2 [18], ensuring full control over data preprocessing, feature extraction, and integration with the LLM-based module. Preprocessing steps for 3D and 2D data, including mesh parsing, image segmentation, and feature computation, were predefined and consistently applied across analyses. The LLM component was incorporated as a downstream module receiving structured inputs derived from these preprocessing steps. The overall computational workflow was executed in a controlled environment to ensure internal consistency and reproducibility; implementation details can be made available upon reasonable request.

### 2.4. LLM-Based Risk Interpretation

After extracting the quantitative descriptors, we used an LLM accessed via the OpenAI API [19] to synthesize the data. The LLM component was implemented in R [18] through a direct HTTPS POST request to the OpenAI Chat Completions endpoint (/v1/chat/completions), using a custom wrapper built with the httr and jsonlite packages rather than a version-pinned SDK [19]. The source code attempted the model alias gpt-5-pro first and gpt-5.1 as a fallback in the event of model availability or access failure [19]. Because date-stamped snapshot identifiers were neither pinned in the request nor logged in the output, the exact historical model version cannot be retrospectively reconstructed with certainty; this should be acknowledged as a limitation for strict reproducibility [20]. The request used a two-message prompt structure. The system prompt assigned the model the role of an “expert paediatric otolaryngologist and biomedical engineer” and instructed it to provide a concise but comprehensive risk assessment of a foreign body entering a child’s airway. The model was explicitly guided to consider the child’s age and sex, the object’s dimensions, shape, and sharpness, the likely site of lodgment, possible complications (including obstruction, mucosal injury, and aspiration), preventive recommendations, evidence-based reasoning, and to avoid overconfidence.

The user prompt concatenated three information blocks:

1. The operator’s free-text description of the object;
2. An automatically generated summary of the object’s quantitative measurements, including 3D volume, surface area, principal axis lengths, elongation, flatness, sphericity, convexity ratio, and 2D silhouette-derived measures such as area, perimeter, major and minor diameters, aspect ratio, circularity, solidity, and minimum curvature radius;
3. Age- and sex-specific approximate normative pediatric airway dimensions for the glottis, subglottis, cricoid, and proximal trachea, reported as anteroposterior diameter, transverse diameter, and cross-sectional area.

The generation parameters explicitly specified in the code were temperature = 0.5 and max_completion_tokens = 5000. Under OpenAI API semantics, temperature controls sampling stochasticity on a 0–2 scale; therefore, a value of 0.5 represents moderate stochasticity and is lower than the default value of 1, favoring more consistent outputs [19,21]. No other generation controls were explicitly set, including top_p, frequency penalty, presence penalty, seed, log probabilities, streaming, structured response format, function or tool calling, or multimodal inputs; accordingly, these parameters should be considered unspecified and governed by API defaults or server-side behavior at runtime [19].

The model generated a narrative risk assessment, comparing object dimensions with airway sizes and inferring likely lodgment sites and complications. The LLM also identified preventive implications and recommended design modifications for similar objects. Although this paper presents a single illustrative case, the same approach can be applied programmatically to all objects in the Susy Safe registry to build a risk stratification tool.

To ensure the plausibility and clinical consistency of the generated interpretations, the LLM outputs were reviewed by four domain experts, two with experience in pediatric otolaryngology and two with experience in airway foreign body management. The expert review focused on verifying the coherence between the quantitative measurements extracted from the 3D reconstruction, the referenced anatomical thresholds, and the resulting clinical interpretation provided by the model. No formal multi-reviewer blinded protocol with quantitative inter-rater agreement was implemented in this proof-of-concept.

### 2.5. Machine Learning Task Definition and Knowledge Extraction Framework

The pipeline can be formalized as a hybrid computational framework combining quantitative feature extraction, rule-based risk evaluation, and knowledge extraction through the LLM.

From a machine learning perspective, the analytical objective of the framework can be interpreted as a risk classification task, where descriptor and physical descriptors extracted from the reconstructed three-dimensional object are evaluated with respect to clinically established anatomical constraints. The goal is to assess the potential clinical risk associated with an ingested foreign body based on its measurable structural characteristics.

The input space of the framework consists of quantitative descriptors derived from the 3D reconstruction process, including principal descriptor dimensions of the object, shape descriptors derived from principal component analysis, and contextual information such as material composition. These descriptors represent the structured feature set used to characterize the object.

Risk evaluation is performed through a rule-based scoring procedure in which extracted measurements are compared with anatomical safety thresholds reported in the clinical literature. Each feature is evaluated against its corresponding threshold to generate a set of binary or categorical risk indicators reflecting whether specific descriptor characteristics may increase the likelihood of airway obstruction, mucosal injury, or lodgment at critical anatomical sites. These indicators are subsequently aggregated to produce an overall qualitative assessment of potential clinical risk.

Within this computational architecture, the LLM is not used as the primary predictive model. Instead, it operates as a knowledge extraction and interpretation layer that integrates structured measurements, threshold-based evaluations, and selected evidence from the scientific literature. Given these inputs, the LLM generates a structured narrative interpretation describing the clinical implications of the observed object characteristics.

This hybrid design separates quantitative measurement and rule-based evaluation from language-based reasoning, allowing the framework to combine transparent feature-driven risk assessment with automated knowledge synthesis derived from large-scale medical literature.

## 3. Results

### 3.1. Morphometric Descriptors of the Case Object

Table 1 summarizes the 3D and 2D descriptors obtained for the metallic object extracted from the child’s airway. The object had a volume of 73.3 mm^3^ and a surface area of 106.3 mm^2^, corresponding to a surface-to-volume ratio A/V of 1.45 mm^−1^. The principal axes indicated moderate anisotropy, with an elongation factor of 0.85 and a flatness of 0.71. The sphericity of 0.80 and convexity ratio of 0.68 demonstrate departure from a perfect sphere and the presence of concave regions. Four edges had dihedral angles below 90°, and the minimum dihedral angle was 13.7°, indicating at least one very sharp projection.

The 2D silhouette area was 3.49 mm^2^ with a perimeter of 7.23 mm. The maximum feret diameter was 2.58 mm and the minimum 1.60 mm, yielding an aspect ratio of 0.62. Circularity (0.84) and solidity (0.99) suggested a roughly compact outline with minimal concavities. The minimum curvature radius of 0.074 mm corresponds to a tip that is extremely sharp relative to the airway epithelium. The object weighs approximately 0.5 g, consistent with a high-density metallic material.

The maximum Feret diameter on the 2D silhouette (2.58 mm) is markedly smaller than the major principal axis derived from the 3D mesh (L1 = 8.24 mm). This is an expected geometric consequence of the two acquisition modalities: the 3D descriptors quantify the object’s maximum extent along arbitrary spatial directions, whereas the 2D Feret diameters quantify only its projection onto the image plane, which depends on the orientation in which the object was placed during photographic acquisition. When comparing object dimensions with airway anatomy, the 3D principal axes therefore provide the more conservative and clinically relevant reference, with the 2D silhouette descriptors offering a complementary perspective.

### 3.2. Comparison with Normative Airway Dimensions

Table 2 compares the object’s principal dimensions with approximate normative airway diameters reported in the radiological literature for a 2-year-old child, used here as an anatomical reference rather than as patient-specific values. The maximum object dimension (8.24 mm) approximates the average anteroposterior diameter of the glottis (~9.8 mm) and approaches the reported subglottic anteroposterior diameter (8.5 mm) [7]. More importantly, the object’s width in the bounding box (6.82 mm) exceeds the narrowest transverse dimension of the glottis (~3.4 mm) and it is comparable to the reported transverse diameter of the cricoid (6.8 mm) [7]. The object’s cross-sectional area (~3.49 mm^2^) is an order of magnitude smaller than the glottic cross-section (26.5 mm^2^) [7], implying that it can traverse the airway if oriented appropriately. However, its irregular shape and sharp tip increase the risk of mucosal injury and lodging. Recommended endotracheal tubes for 2-year-olds have internal diameters of 4.0–4.5 mm and outer diameters of 5.3–6.0 mm [17]; the object’s minor dimension (4.5 mm) would match the internal diameter and could obstruct the tube. Pediatric airway dimensions are known to vary substantially between individuals at any given age depending on sex, body size, and individual anatomy; the comparison reported here should therefore be interpreted as an approximate anatomical reference rather than as a patient-specific evaluation.

### 3.3. Risk Stratification from Literature

Table 3 synthesizes literature findings on how object shape affects lodging site and injury severity. These studies provide the clinical context used in the proposed workflow to interpret the morphometric descriptors of the analyzed object. The Susy Safe 3D-scanning study found that spherical objects with low surface friction were associated with tracheal or bronchial lodgment (odds ratio 2.8; 95% CI 1.9–4.2). Irregular or angular objects with sharp projections were associated with esophageal impaction (odds ratio 3.1; 95% CI 2.0–4.9) [11]. A retrospective clinical study emphasized that slimmer, sharper objects tend to travel deeper down the bronchial tree and become dangerous if they reach the cricoid membrane [5]. Automated 2D shape classification achieved about 72% accuracy in recognizing silhouettes as circular, polygonal, sharp or irregular, and location classification accuracy of 88% [22], suggesting that computer vision can provide useful descriptors for risk assessment.

### 3.4. Large Language Model Interpretation

The LLM evaluated the descriptors in Table 1, Table 2 and Table 3 to infer aspiration risk. It noted that the object’s maximum dimension is similar to the glottic anteroposterior diameter, whereas its width exceeds the narrow transverse diameter; the object could therefore align longitudinally to pass through the glottis but may lodge if rotated. The sphericity of 0.80 and convexity ratio of 0.68 suggest an irregular shape rather than a smooth sphere; combined with the extremely sharp tip (curvature radius 0.074 mm), the object likely behaved as an irregular or angular foreign body in the classification scheme. According to Table 3, such objects tend to lodge in the esophagus and carry a higher risk of laceration [11]. The LLM also emphasized that the cross-sectional area of the object (3.49 mm^2^) is much smaller than typical airway cross-sections, meaning that it can traverse the airway but may cause deep injury if oriented vertically. The moderate elongation further implies that it can wedge diagonally. Finally, the LLM recommended that objects with similar morphometric profiles be flagged in product safety databases and that clinicians exercise caution during extraction, grasping the sharp end to avoid mucosal damage [23].

## 4. Discussion

### 4.1. Interpretation of Case Findings

The case object illustrates how quantitative descriptors enrich clinical understanding of FBA. Although the object’s volume (73 mm^3^) and projected area (3.49 mm^2^) are well below the airway cross-section, its maximum length (8.2 mm) nearly matches the glottic anteroposterior diameter. It could therefore traverse the glottis if oriented along its long axis, but its width (6.8 mm) exceeds the narrowest glottic transverse dimension (~3.4 mm) [7]. The object is moderately elongated and flattened; in contrast to smooth spheres that may slide into the distal bronchi, its irregular shape and sharp edge increase friction and risk of tissue damage. According to Susy Safe modelling results, irregular/angular objects are more likely to impact the esophagus [11], whereas spherical objects lodge in the trachea. The LLM predicted that the object might have wedged at the cricoid cartilage before passing into the trachea; indeed it was recovered from the laryngo-tracheal tract, indicating partial obstruction and potential mucosal injury.

Existing computational approaches for risk assessment in medical imaging, including radiomics and computer vision pipelines, typically focus on extracting quantitative descriptors and using statistical or machine learning models to predict outcomes. Recent studies have explored the use of machine learning and deep learning models for pediatric airway triage and foreign body aspiration risk assessment [24,25,26]. These approaches include multimodal AI systems and deep learning–based analysis of medical imaging, and reported results highlight both the potential and the current limitations of purely predictive AI models in complex clinical scenarios. In contrast, the framework proposed here combines quantitative descriptor feature extraction with a knowledge extraction layer based on LLM. In this architecture, the LLM does not act as a predictive model but as an interpretative component that synthesizes structured measurements and relevant literature to produce a clinically contextualized risk interpretation.

From a methodological perspective, the main contribution of this work lies in the integration of quantitative descriptor feature extraction with an LLM used as a knowledge extraction layer within the proposed computational workflow, enabling structured measurements to be translated into interpretable risk-oriented insights.

### 4.2. Integration of 2D/3D Morphometry with LLM Reasoning

This work demonstrates a novel integration of morphometric analysis and LLM-based synthesis, based on three complementary components. First, high-resolution digitization enables detailed characterization of foreign bodies: 3D scanning captures subtle features such as concave indentations and sharp projections that are not apparent on standard 2D radiographs, while calibrated 2D images provide rapid silhouette-based screening when 3D acquisition is unavailable. Second, quantitative morphometric descriptors allow systematic comparison across objects and linkage with clinical outcomes. Standardized descriptors such as volume, surface area, sphericity, elongation, convexity, and dihedral angles capture size, shape irregularity, and surface complexity; radiomics research has shown that sphericity values close to 1 indicate rounded objects, whereas lower values reflect increasing irregularity [9,10], while aspect ratio and circularity quantify how elongated or irregular the silhouette is [15]. Third, LLM-based interpretation integrates these quantitative features with contextual information such as normative airway dimensions and clinical guidelines, generating an explanatory narrative for each case. This ability to reason across heterogeneous data sets LLMs apart from traditional machine-learning classifiers. Compared with conventional machine learning approaches, LLMs can integrate heterogeneous sources of information, including structured measurements, clinical guidelines and published literature, and translate them into interpretable narrative explanations. However, unlike predictive models trained on labeled datasets, LLM outputs may depend on prompt formulation and may occasionally generate inaccurate statements. In our example, the LLM integrated normative airway sizes, Susy Safe risk statistics and the object’s descriptors to infer that the object is likely to lodge proximally and cause mucosal injury if oriented unfavourably.

As a proof-of-concept, the proposed framework was implemented in an interactive R Shiny [27] prototype that allows exploratory integration of object morphometrics, pediatric airway reference dimensions, and LLM-generated narrative summaries (Appendix B).

The use of LLMs in clinical reasoning workflows also raises important reliability considerations. LLMs are known to occasionally generate plausible but incorrect statements (so-called hallucinations), particularly when synthesizing information from heterogeneous sources. In the present framework this risk is mitigated by grounding the model input in structured quantitative measurements and explicitly defined anatomical thresholds derived from the literature. Nevertheless, the generated interpretations should be considered as decision-support outputs rather than autonomous clinical judgments, and expert verification remains an essential component of the workflow.

### 4.3. Implications for Prevention and Policy

Quantitative assessment of object morphology could transform injury prevention. Many safety standards rely on size thresholds alone, such as the small-parts test cylinder that determines whether a toy component is a choking hazard. Our analysis shows that shape and surface features also influence risk: slender, sharp objects may pass the size test but still endanger the airway [5]. By scanning consumer products and computing these descriptors, regulators could develop shape-aware guidelines. For example, packaging could display a hazard label if the product’s sphericity is below a threshold or if it contains edges with dihedral angles below 90°. Manufacturers could redesign products to increase sphericity or blunt sharp points, reducing the odds of lodging or laceration.

Integrating morphometric data into public databases such as Susy Safe will also enhance surveillance. Papappicco et al. proposed a web-based platform where clinicians can upload scans and receive real-time risk assessments [11]. Coupling such a platform with LLMs could provide intuitive explanations for parents and healthcare workers, bridging the gap between quantitative analysis and prevention messaging. Education campaigns can emphasize that not only small size but also irregular shape and sharpness increase danger; this is particularly important in rural or low-resource settings where awareness is low [1,5].

### 4.4. Clinical Relevance for the Practicing Pediatrician

Although the present work is methodological in nature, its rationale and intended use are firmly grounded in everyday pediatric practice. For the emergency physician, the most immediate implication is that small-part size tests do not capture the full hazard profile of an object: a fragment may pass the test cylinder yet, by virtue of an irregular profile or a sharp tip, still cause mucosal laceration, lodge at the cricoid, or migrate distally into a main bronchus. The morphometric descriptors discussed here—elongation, sphericity, convexity, and minimum curvature radius—formalize features that experienced clinicians already recognize intuitively at the bedside, and provide a common vocabulary that can be shared with radiologists, otolaryngologists, and product-safety authorities. For the primary-care pediatrician, the same descriptors translate into concrete caregiver-education messages: beyond size alone, features such as “sharp”, “elongated”, or “irregular” should trigger removal of an object from a young child’s environment. Finally, the framework does not require complex infrastructure: a calibrated smartphone photograph of a retrieved object is sufficient to obtain the 2D descriptor set, making the approach feasible even in resource-limited emergency departments. In this sense, the computational component is a means to an end—the end being safer products, better-informed caregivers, and more accurate clinical risk stratification.

### 4.5. Limitations and Future Directions

This study has limitations. Only one object was analyzed as a case study; while it demonstrates feasibility, broader generalization requires analysis of many objects across different materials and shapes. Our 3D scanning and imaging were performed ex vivo; shape or size may change during aspiration due to compression or swelling. The LLM’s reasoning is based on available literature and may not capture all biomechanical factors, such as airflow patterns or mucosal elasticity. The specific 3D scanner model and firmware version, as well as the verbatim raw output produced by the LLM for the present case object, were not preserved in machine-readable form at the time of acquisition; this affects strict reproducibility, although the structured input prompt, generation parameters, and software pipeline are fully described in Section 2.4. Automated shape classification accuracy is still moderate (~72%) [22]; further improvements and validation are needed. Future research should incorporate dynamic simulations, material properties (e.g., friction coefficient) and prospective clinical validation. Nevertheless, combining morphometric descriptors with LLM reasoning holds promise for developing decision-support tools that aid physicians and inform safer product design.

The present study should therefore be considered primarily as a proof-of-concept demonstration of the proposed computational workflow rather than a large-scale validation study. The pipeline is illustrated through a representative case to demonstrate how 3D descriptor reconstruction, rule-based risk indicators, and LLM-based knowledge synthesis can be integrated within a single analytical framework. A systematic evaluation across a larger dataset of foreign body objects would represent an important next step. In particular, applying the pipeline to a broader collection of objects from registries such as Susy Safe could allow quantitative assessment of robustness, consistency, and agreement with expert clinical evaluation across different object morphologies. Such large-scale validation is planned as part of future work.

## 5. Conclusions

Foreign-body aspiration remains a preventable cause of morbidity and mortality in children. Quantitative 2D and 3D morphometry provides objective information on an object’s shape, size, and surface features, which—when interpreted in light of normative airway dimensions and clinical evidence—may contribute to a more informed assessment of likely lodgment patterns and potential injury severity. LLMs offer a new complementary way to synthesize these diverse data sources into interpretable risk assessments. The case study presented here illustrates, as a proof-of-concept, that a small metallic object with moderate elongation, but a sharp tip, can traverse the airway but may injure mucosa or lodge depending on orientation. Integrating such analyses into surveillance registries and regulatory frameworks could enhance prevention by flagging dangerous designs, guiding public health messaging and supporting clinicians in managing foreign body aspiration.

## Figures and Tables

**Table 1:** 3D and 2D morphometric descriptors of the case object.

Descriptor	Value	Interpretation
Volume (mm^3^)	73.3	Small; fits within the throat test cylinder, indicating potential to enter airway.
Surface area (mm^2^)	106.3	Combined with volume yields a surface-to-volume ratio A/V ≈ 1.45 mm^−1^, indicating moderate surface exposure.
Principal dimensions (mm)	$L_{1}=8.24$ $L_{2}=5.72$ $L_{3}=4.51$	Maximum dimension comparable to glottic diameter (~9 mm); minor dimensions smaller than subglottic widths.
Bounding box width (mm)	6.82	Width of the minimum axis-aligned bounding box of the 3D mesh along the second principal axis.
Elongation	0.85	Moderate elongation; object is neither very rod-like nor spherical.
Flatness	0.71	Somewhat flattened shape.
Sphericity	0.80	Departures from sphere reflect irregularity.
Convexity ratio (volume/hull volume)	0.68	Presence of concave indentations on the surface.
Sharp edges (dihedral < 90°)	4 edges. Minimum angle 13.7°	Presence of a very sharp projection may injure mucosa.
2D area (mm^2^)	3.49	Small cross-section relative to pediatric airway cross-sections (26–40 mm^2^).
Perimeter (mm)	7.23	Overall outline length of the silhouette.
Max/Min Feret diameters (mm)	2.58/1.60	Cross-sectional dimensions of the object along its longest and shortest directions.
Aspect ratio	0.62	Moderately elongated silhouette.
Circularity	0.84	Near 1 for circle; indicates fairly regular outline.
Solidity	0.99	Contour is almost convex.
Minimum curvature radius (mm)	0.074	Extremely sharp tip that may puncture the airway mucosa.

**Table 2:** Comparison of case object dimensions with approximate normative airway dimensions reported in the radiological literature for a 2-year-old child, used as anatomical reference values rather than as patient-specific measurements.

Parameter	Typical Value (mm)	Source & Remarks	Case Object (mm)
Glottic anteroposterior diameter	9.8 ± 1.1	Radiography study (≤4 year) [7]	8.24 (L_1_)
Glottic transverse diameter	3.4 ± 0.9	Radiography study [7]	6.82 (bounding box width)
Glottic area	26.5 mm^2^	Radiography study [7]	3.49 mm^2^
Subglottic anteroposterior diameter	8.5 ± 0.6	Radiography study [7]	8.24
Subglottic transverse diameter	5.6 ± 0.8	Radiography study [7]	5.72
Cricoid anteroposterior × transverse diameter	7.4 × 6.8	Radiography study [7]	8.24 × 6.82
Proximal trachea diameter (median)	7.3 (boys), 6.5 (girls)	Ultrasonography [17]	8.24
Endotracheal tube internal diameter	4.0–4.5	Guideline for 2-year olds [17]	4.51 (minor dimension)

**Table 3:** Literature evidence relating object shape to lodging and risk.

Finding	Evidence	Citation
Spherical, low-friction objects lodge in trachea/bronchi	Among 500 scanned foreign bodies, spherical shapes with low surface friction were more likely to lodge in the trachea or bronchi (OR 2.8; 95% CI 1.9–4.2).	Papappicco et al. 2025 [11]
Irregular or angular objects with sharp projections lodge in esophagus	Irregular or angular shapes were associated with esophageal lodgment (OR 3.1; 95% CI 2.0–4.9).	Papappicco et al. 2025 [11]
Slim, sharp objects travel deeper into bronchial tree	Retrospective study noted that slimmer, sharper foreign bodies tend to travel further down the bronchial tree and become dangerous if they reach the cricoid membrane.	Mîndru et al. 2023 [5]
Automated shape classification accuracy	A radiographic analysis reported that automatic shape determination achieved ~72% accuracy in classifying foreign body silhouettes (circle, polygon, sharp, irregular) and 88% accuracy in predicting lodgment location.	Vasumathy et al. 2019 [22]

**Figure A3:**
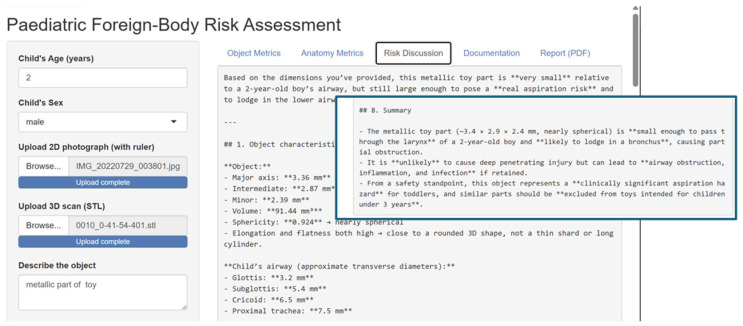
Graphical interface of the R Shiny research prototype illustrating the exploratory integration of object morphometric descriptors, pediatric airway reference dimensions, and an LLM-generated explanatory narrative.

## Data Availability

The dataset is available on motivated request to the corresponding author.

## Abbreviations

The following abbreviations are used in this manuscript:

FBA	Foreign-body aspiration
CT	Computed tomography
LLM	Large language model
STL	Stereolithography
PCA	Principal component analysis
MR	Magnetic resonance
CI	Confidence interval
3D	Three-dimensional
2D	Two-dimensional

## Appendix A

3D morphometric features were extracted from triangulated STL surface meshes obtained through structured-light scanning of the retrieved object (Figure A1). The 3D representation enabled computation of volumetric properties and rotation-invariant shape descriptors, capturing overall object size, elongation, and surface irregularity.

2D morphometric features were derived from calibrated photographic images acquired with a descriptor reference (Figure A2). Following silhouette extraction, planar shape descriptors were computed to characterize object outline and cross-sectional properties. These 2D features offer a complementary representation of object geometry, particularly relevant in contexts where only projection-based information is available.

Together, the combined 2D and 3D morphometric descriptors provide a consistent quantitative characterization of object shape and size. These features were subsequently interpreted in relation to pediatric airway dimensions and integrated within the large LLM–based reasoning framework described in the main text.

## Appendix B. Interactive R Shiny Prototype

An interactive prototype was developed using the R Shiny [27] framework to demonstrate the practical integration of morphometric features, anatomical reference data, and LLM–based reasoning within the proposed risk assessment approach (Figure A3).

The prototype allows users to input basic patient characteristics (age and sex) and to upload either a calibrated two-dimensional photograph or a three-dimensional STL model of a foreign body. The application computes object-level morphometric descriptors consistent with those described in the main Methods section and displays them in tabulated form. In parallel, age- and sex-specific pediatric airway reference dimensions are presented to provide anatomical context.

Based on the combined object descriptors and airway reference values, the prototype generates an explanatory, text-based risk discussion intended to illustrate how quantitative measurements can be translated into an interpretable narrative. This narrative output is designed for research and exploratory purposes only and does not constitute clinical decision support.

The R Shiny application is provided as a demonstrative research prototype to illustrate potential translational implementations of the proposed framework. It has not undergone clinical validation and is not intended for diagnostic or therapeutic use.
